# Engineering CAR-T cells for solid tumors: overcoming antigenic, trafficking, and microenvironmental barriers

**DOI:** 10.3389/fimmu.2026.1891408

**Published:** 2026-07-29

**Authors:** Ziyan Kong, Jinke Wang

**Affiliations:** School of Biological Science and Medical Engineering, Southeast University, Nanjing, China

**Keywords:** antigen heterogeneity, armored CAR-T cells, CAR-T cell therapy, solid tumors, synthetic receptors, tumor microenvironment

## Abstract

Chimeric antigen receptor T-cell (CAR-T) therapy has demonstrated remarkable clinical efficacy across a spectrum of hematological malignancies, with particularly profound responses observed in B-cell leukemias, lymphomas, and multiple myeloma. However, the clinical benefits of CAR-T cell therapy have not yet been effectively extended to most solid tumors. This limitation arises from multiple biological and structural barriers, including the paucity of truly tumor-specific antigens, variable antigen expression and loss, inefficient trafficking and infiltration, and the presence of a highly immunosuppressive tumor microenvironment (TME). These obstacles not only restrict tumor recognition and intratumoral accumulation but also impair CAR-T-cell persistence, cytotoxicity, and durable tumor control, while promoting immune escape and increasing the likelihood of on-target, off-tumor toxicity. To address these challenges, diverse engineering strategies are being developed to improve the safety, efficacy, and adaptability of CAR-T cell therapy in solid tumors. These include Boolean logic-gated receptors, multi-antigen and retargeting platforms, locoregional delivery and trafficking-enhancing approaches, checkpoint blockade, armored CAR-T cells, synthetic receptors that rewire inhibitory signals, and emerging *in vivo* engineering approaches. In this review, we discuss how these next-generation engineering strategies are being designed to overcome the obstacles that constrain CAR-T-cell efficacy in solid tumors and to guide the development of safer and more effective therapeutic platforms.

## 1 Introduction

**Figure 1:**
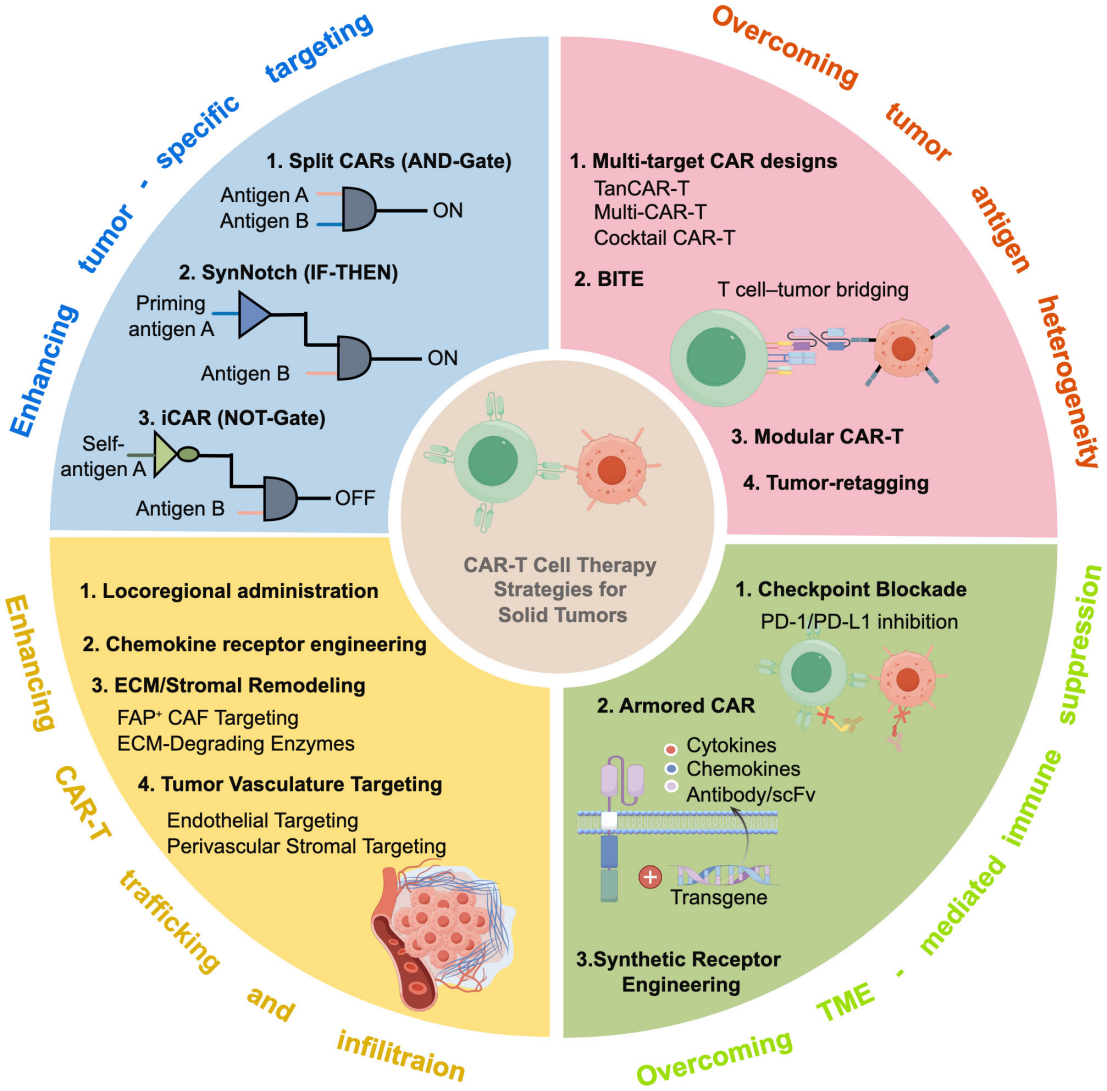
Overview of engineering strategies to enhance CAR-T-cell therapy in solid tumors. Solid tumors impose multiple barriers to effective CAR-T-cell therapy, including limited tumor-specific antigen expression, antigen heterogeneity, insufficient trafficking and infiltration, and an immunosuppressive tumor microenvironment. The figure summarizes four major strategy categories designed to address these barriers. Tumor-specific targeting can be improved through split CARs, synNotch circuits, and inhibitory CARs, which use AND-gate, IF–THEN, or NOT-gate logic to refine tumor recognition. Antigen heterogeneity can be addressed by multi-target CAR designs, BiTE-mediated T cell–tumor bridging, modular CAR-T platforms, and tumor-retagging approaches. CAR-T-cell trafficking and tumor infiltration can be enhanced by locoregional administration, chemokine receptor engineering, ECM/stromal remodeling, and tumor vasculature targeting. TME-mediated immune suppression can be counteracted by PD-1/PD-L1 checkpoint blockade, armored CAR-T cells, and synthetic receptor engineering. These complementary strategies aim to enhance tumor selectivity, improve tumor access, restore CAR-T-cell function, and increase therapeutic efficacy in solid tumors. The figure was created with Figdraw (https://www.figdraw.com).

Chimeric antigen receptor T-cell (CAR-T) therapy has become a major advance in the management of hematological malignancies (1). Durable responses to CD19-directed CAR-T cells in relapsed or refractory B-cell malignancies, together with the substantial clinical activity of B-cell maturation antigen (BCMA)-targeted CAR-T cells in heavily pretreated multiple myeloma, have established this strategy as an important therapeutic modality (2). Currently, seven CAR-T cell products have been approved by the U.S. Food and Drug Administration (FDA), all for hematological malignancies, whereas no CAR-T therapy has yet been approved for solid tumors (3). In solid tumors, the paucity of truly tumor-specific antigens remains a major barrier to CAR-T-cell therapy, as most candidate targets are tumor-associated antigens (TAAs) that are also expressed, to varying degrees, in normal tissues (4). This overlap narrows the therapeutic window and can result in serious on-target, off-tumor toxicity, as illustrated by severe pulmonary toxicity after human epidermal growth factor receptor 2 (HER2)-directed CAR-T-cell infusion and hepatobiliary toxicity associated with carbonic anhydrase IX (CAIX) recognition on bile duct epithelium (5, 6). At the same time, heterogeneous antigen expression and therapy-driven antigen loss can compromise efficacy by allowing tumor subclones to escape CAR-T-cell–mediated killing (7). Beyond antigen recognition, inefficient trafficking to tumor sites and limited infiltration into the tumor parenchyma further restrict the therapeutic activity of CAR-T cells in solid tumors (8–10). This process is often impaired by mismatched chemokine–receptor axes, altered adhesion-molecule expression, and disorganized tumor vasculature, all of which limit T-cell homing and intratumoral entry (11). In addition, a dense extracellular matrix and cancer-associated fibroblast-rich desmoplastic stroma, frequently marked by fibroblast activation protein-α (FAP), form physical barriers that restrict CAR-T-cell penetration and intratumoral accumulation (12). Once CAR-T cells enter the tumor bed, their activity is further constrained by the immunosuppressive tumor microenvironment (TME), which integrates inhibitory checkpoint signaling, suppressive cytokines, regulatory cell populations, and metabolic stress to impair T-cell proliferation, persistence, and cytotoxicity (13, 14). These multilayered barriers indicate that effective CAR-T therapy for solid tumors will require integrated engineering strategies. As summarized in **Figure 1**, current engineering approaches mainly aim to enhance tumor-selective recognition, mitigate antigen escape, improve CAR-T-cell trafficking and tumor infiltration, and counteract TME-mediated immune suppression. In this review, we discuss next-generation strategies addressing these challenges, with particular emphasis on logic-gated receptors, multi-antigen and retargeting platforms, trafficking-enhancing approaches, armored CAR-T cells, synthetic receptors that rewire inhibitory signals, and recently developed approaches for generating CAR-T cells directly *in vivo*, as summarized in **Table 1**.

**Table 1:** Comparative summary of major next-generation CAR-T engineering strategies for solid tumors.

Major strategy	Representative approaches	Main advantages	Key limitations and safety considerations	Clinical/translational status
Logic-gated and microenvironment-responsive CAR systems	Split CARs, synNotch, iCARs, hypoxia-responsive CARs	Improve tumor selectivity by requiring antigen combinations or tumor-associated microenvironmental cues	Activity may be reduced in antigen-heterogeneous tumors; synNotch systems may show delayed activation or leakage	Mostly preclinical; selected synNotch- or hypoxia-regulated CAR-T platforms have entered early clinical evaluation
Multi-target CARs	Tandem CARs, multi-CARs, cocktail CAR-T	Broaden antigen coverage; mitigate antigen escape via OR-gate logic	Broader antigen recognition may increase on-target, off-tumor toxicity if selected antigens are not tumor-restricted	Preclinical to early clinical development; several dual- or multi-target CAR-T products are under clinical testing
Modular and adaptor-based CAR platforms	UniCAR, SUPRA CAR	Enable switchable, titratable, and multi-antigen targeting without re-engineering T cells	Depend on repeated adaptor administration; adaptor pharmacokinetics and immunogenicity may affect sustained activity and safety	Largely preclinical, with selected platforms progressing toward clinical translation
BiTE-secreting CAR-T platforms	BiTE-secreting CAR-T cells; CAR-T combined with BiTE-expressing vectors	Redirect CAR-T cells and bystander T cells to additional tumor antigens; may overcome antigen heterogeneity	Local or systemic BiTE exposure may increase cytokine release or off-tumor T-cell activation	Preclinical to early clinical development, including first-in-human studies in glioblastoma
Antigen retagging	Oncolytic viruses, mRNA/LNP, amphiphiles, bacterial vectors	Bypass dependence on endogenous tumor antigens and render antigen-negative tumors targetable	Delivery-system toxicity, viral/bacterial safety, antigen spread, and residual antigen-negative foci require evaluation	Mainly preclinical, with clinical translation remaining to be established
Trafficking- and infiltration-enhancing strategies	Locoregional delivery, chemokine receptor engineering, ECM or stromal remodeling, vascular targeting	Enhance tumor homing and intratumoral infiltration	Aberrant homing, stromal toxicity, vascular damage, and local inflammatory toxicity should be monitored	Locoregional delivery has entered clinical testing; most chemokine-, stromal-, and vascular-targeting approaches remain preclinical or early clinical
Checkpoint-resistant CAR-T strategies	Checkpoint blockade combination, PD-1 or CTLA-4 knockdown/knockout, CAR-T-secreted checkpoint-blocking scFvs	Restore CAR-T activity in checkpoint-rich TMEs and may improve persistence	May not overcome non-checkpoint suppressive barriers; added engineering or combination therapy may increase immune-related toxicity	Preclinical to early clinical development; selected checkpoint-modulated CAR-T strategies have entered clinical testing
Armored CAR-T	CAR-T cells secreting cytokines, checkpoint-blocking antibodies, or other immunomodulatory payloads	Enhance CAR-T persistence, proliferation, and endogenous antitumor immunity; remodel TME	Payload expression must be tightly controlled to avoid exhaustion or systemic toxicity	Preclinical to early clinical development, with selected cytokine-armored CAR-T cells evaluated in clinical trials
Synthetic receptors that rewire inhibitory signals	Chimeric switch receptors, inverted cytokine receptors, dominant-negative receptors	Convert suppressive checkpoint or cytokine signals into activating or pro-survival cues	Efficacy and safety depend on ligand abundance and spatial distribution; off-tumor ligand expression may cause unintended activation	Predominantly preclinical
*In vivo* CAR-T cell generation platforms	T-cell-targeted lentiviral vectors, AAV-based delivery systems, mRNA/LNP platforms	Simplify manufacturing and enable direct programming of endogenous T cells	Delivery specificity, expression durability, repeat dosing, off-target delivery, and long-term safety remain unresolved	Preclinical to early clinical development; selected lentiviral and mRNA/LNP candidates have entered phase I trials

AAV, adeno-associated virus; BiTE, bispecific T-cell engager; CAR, chimeric antigen receptor; CTLA-4, cytotoxic T lymphocyte-associated protein 4; ECM, extracellular matrix; iCAR, inhibitory CAR; LNP, lipid nanoparticle; mRNA, messenger RNA; PD-1, programmed cell death protein 1; scFv, single-chain variable fragment; synNotch, synthetic Notch; TME, tumor microenvironment; UniCAR, universal CAR; SUPRA CAR, split, universal, and programmable CAR. Clinical/translational status is summarized based on representative examples discussed in the corresponding sections and is not intended as an exhaustive clinical trial registry.

## 2 Enhancing tumor-specific targeting

**Figure 2:**
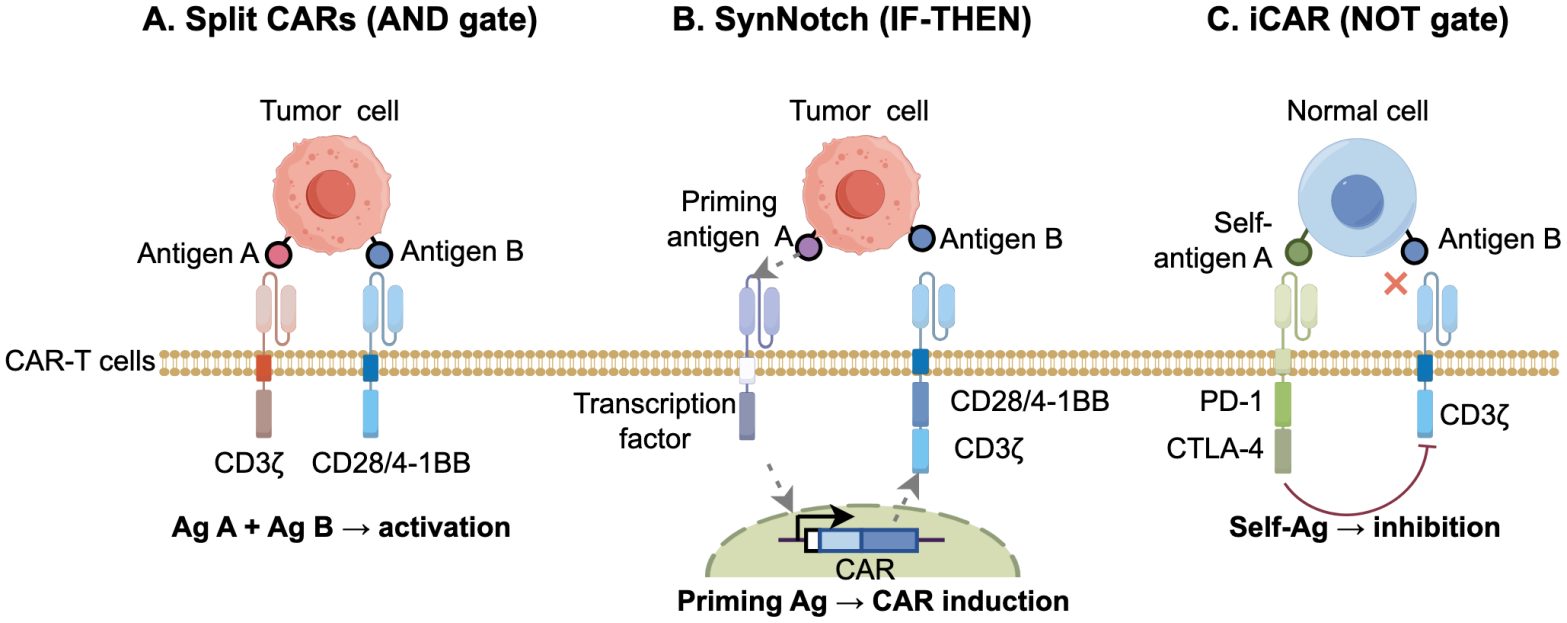
Boolean logic-gated CAR-T-cell designs for improving tumor-specific targeting. **(A)** Split CARs implement an AND-gate strategy by separating CD3ζ-mediated activation and co-stimulatory signaling into two receptors, so that full CAR-T-cell activation occurs preferentially when tumor cells co-express both antigens. **(B)** SynNotch circuits function as an IF–THEN system, in which recognition of a priming antigen induces transcriptional activation and subsequent CAR expression against a second tumor-associated antigen. **(C)** Inhibitory CARs (iCARs) establish a NOT-gate mechanism by recognizing antigens expressed on normal tissues and delivering inhibitory signals through PD-1- or CTLA-4-derived intracellular domains, thereby limiting CAR-T-cell activation against normal cells. The mechanism labels highlight the core logic of each design: dual-antigen recognition promotes activation, priming-antigen recognition induces CAR expression, and self-antigen recognition triggers inhibition. These logic-gated designs improve discrimination between malignant and normal tissues and may reduce on-target, off-tumor toxicity. The figure was created with Figdraw (https://www.figdraw.com).

A major obstacle for CAR-T-cell therapy in solid tumors is the scarcity of truly tumor-specific antigens (TSAs). Most targets currently under investigation are tumor-associated antigens (TAAs), which are also expressed at variable levels in normal tissues, thereby limiting the margin between efficacy and toxicity and increasing the likelihood of on-target, off-tumor effects. Clinical experience has illustrated this limitation across both hematologic malignancies and solid tumors. CD19-directed CAR-T cells may require long-term immunoglobulin replacement and have been associated with neurotoxicity-related concerns, whereas CAIX-, HER2-, mesothelin-, Claudin 18.2-, and CEACAM5-directed CAR-T therapies have produced hepatobiliary, pulmonary, gastrointestinal, or respiratory toxicities due to antigen expression in normal tissues (5, 15–19). Taken together, these findings underscore the need to balance antitumor potency with safety, particularly when target antigens are co-expressed by cancer cells and normal tissues. To address this challenge, Boolean logic–gated receptor designs have emerged as a more precise strategy to improve tumor specificity by restricting T-cell activation to defined antigenic conditions, building on early efforts to engineer multi-receptor CAR-T systems that respond to combinations of antigens (20). Representative AND-, IF–THEN-, and NOT-gate CAR-T designs are illustrated in **Figure 2**.

Within this framework, split CAR systems distribute activation (CD3ζ) and co-stimulatory signals (e.g., CD28 or 4-1BB) across two independent receptors recognizing distinct antigens, such that full T-cell activation requires the simultaneous engagement of both targets, thereby forming an AND-gate. This combinatorial requirement enhances tumor specificity and reduces toxicity toward normal tissues by restricting cytotoxic responses to cells co-expressing both antigens (21). Kloss et al. paired an anti-CD19 activation receptor with a PSMA-targeted co-stimulatory receptor, demonstrating that split CAR-T cells could mediate effective tumor control in dual-antigen-positive xenograft models (21). In a related approach, Lanitis et al. separated CD3ζ and CD28 signaling into two CARs with distinct antigen specificities, targeting mesothelin and folate receptor-α (FRα), respectively. This trans-signaling design retained potent antitumor activity against dual-antigen-positive tumors while reducing responses to single-antigen-positive cells that modeled normal tissues (22). More recently, related principles of combinatorial control have also been extended to modular adaptor-based platforms, such as the reverse chimeric antigen receptor (RevCAR) system, which enables tunable and combinatorial antigen recognition through bispecific targeting modules (23). However, these studies also highlight an important limitation of classical split CAR designs: signal 1–only CARs may retain residual activity and mediate cytotoxicity in the absence of co-stimulation, thereby permitting the killing of single-antigen-expressing cells and compromising specificity. To partially mitigate this limitation, affinity tuning has been explored as an optimization strategy within split CAR systems. Specifically, this design can pair a low-affinity activation receptor recognizing a widely expressed target with a high-affinity co-stimulatory receptor recognizing a more tumor-selective antigen, thereby raising the activation threshold and improving selectivity. Consistent with this approach, a CD38/CD138 split CAR design demonstrated effective antitumor activity in multiple myeloma models without damaging normal cells expressing only one of the two target antigens (24).

In settings where off-target tissues are spatially separated from tumor cells, sequential activation strategies can be leveraged to enhance targeting specificity. Synthetic Notch (synNotch) receptor systems have therefore been developed to support sequential antigen-gated CAR-T-cell activation, whereby engagement of an initial antigen induces expression of a second-target CAR (25–27). Mechanistically, CAR-T cells are engineered with two genetic modules: a constitutively expressed synNotch receptor and a CAR construct driven by a synNotch-responsive promoter. Upon engagement with the priming antigen, synNotch activation releases an intracellular transcriptional activator that drives CAR expression, thereby establishing a sequential (IF–THEN) gating logic that restricts effector function to defined antigenic contexts (28). Recent studies have established synNotch-based platforms capable of implementing multi-antigen “prime-and-kill” circuits, in which recognition of a priming antigen induces CAR expression against a second tumor-associated antigen (28, 29). This strategy has been applied across different antigen combinations and tumor contexts, including EGFRvIII-gated CAR induction in glioblastoma, CD33-gated CD123 CAR expression in acute myeloid leukemia, and ROR1- or dual-antigen-based circuits designed to reduce off-tumor toxicity (28–32). In addition to improving specificity, inducible CAR expression may also reduce tonic signaling, thereby supporting metabolic fitness, persistence, and resistance to exhaustion in synNotch CAR-T systems (29). Together, these findings support synNotch circuits as flexible platforms for spatially restricted and sequentially controlled CAR-T-cell activation. Nevertheless, AND-gated and synNotch CAR-T systems require coordinated recognition of a defined antigen pair, and incomplete co-expression may restrict their activity in heterogeneous solid tumors. Hernandez-Lopez et al. demonstrated that HER2 synNotch signaling has an ultrasensitive antigen-density threshold, with low-density HER2 producing weak CAR induction and high-density HER2 driving robust CAR expression and effector activity (33). Moreover, because synNotch-gated CAR induction requires transcription and protein synthesis, cytotoxic licensing is delayed and becomes transient after withdrawal of the priming antigen, as reflected by the approximate 8 h half-life of induced CAR expression reported by Roybal et al. (25). Another practical concern is ligand-independent activation or transcriptional leakage, whereby basal synNotch receptor cleavage and/or low-level background payload expression may induce transgene expression even in the absence of priming-antigen engagement, thereby weakening antigen-gated specificity (34). Together, these threshold-, kinetic-, and leakage-related constraints indicate that synNotch circuits involve a tradeoff between improved specificity and delayed or imperfectly gated effector activation, which may permit tumor escape or off-target activity in highly heterogeneous tumors.

Another strategy involves inhibitory CAR (iCAR) constructs, which function as NOT-gate systems by recognizing antigens expressed on normal tissues. In this approach, CAR-T cells are engineered to express receptors containing inhibitory signaling domains derived from PD-1 or CTLA-4. Upon antigen engagement, these receptors transmit dominant inhibitory signals that suppress T-cell activation (35). This veto-like mechanism prevents cytotoxic responses against healthy cells, thereby providing an additional safety layer to limit on-target, off-tumor toxicity (36–38).

Tumor microenvironment-derived characteristics, such as hypoxia, can also be exploited to improve the spatial selectivity of CAR-T cell activation. One early strategy fused an oxygen-dependent degradation domain (ODD) derived from hypoxia-inducible factor-1 alpha (HIF-1α) to the CAR scaffold, promoting CAR degradation under normoxic conditions and stabilization under hypoxia (39). Although this approach enhanced hypoxia-dependent tumor-cell killing, residual activity under normoxic conditions indicated incomplete regulation (39). Later designs combined transcriptional and post-translational oxygen sensing to establish a dual oxygen-sensing strategy. For example, HypoxiCAR incorporates hypoxia-responsive elements (HREs) into the CAR expression cassette together with an ODD-modified CAR, enabling stringent hypoxia-specific CAR expression that prevents on-target, off-tumor activation while maintaining potent antitumor activity (40). More recently, hypoxia-responsive CEA-targeted CAR-T cells have entered phase I clinical evaluation (NCT05396300). Preliminary clinical data suggest a higher objective response rate (ORR) in the intraperitoneal (I.P.) cohort (25%) compared with the intravenous (I.V.) cohort (8%) (41). However, partial overlap between tumor hypoxia and physiological or pathological low-oxygen niches in normal tissues underscores the importance of accounting for tissue oxygen heterogeneity in CAR design to minimize potential off-tumor effects (42, 43). Taken together, antigen-based logic-gated CAR designs and TME-responsive control systems provide complementary frameworks for improving tumor specificity in solid tumors. By integrating activating, sequential, inhibitory, or microenvironment-derived regulatory cues, these strategies enable more precise spatial and antigenic discrimination between malignant and normal tissues while preserving antitumor activity.

## 3 Overcoming tumor antigen heterogeneity

One major obstacle for CAR-T therapy in solid tumors is the absence of broadly and consistently expressed targets comparable to clinically exploited antigens in hematologic malignancies (44). Most candidate targets in solid tumors are TAAs, whose heterogeneous expression, limited surface density, and dynamic downregulation or loss during tumor evolution substantially compromise CAR-T cell recognition and antitumor efficacy (7, 45, 46). To address tumor antigen heterogeneity and therapy-driven immune escape, multiple therapeutic approaches have been developed, including multi-antigen CAR designs, modular or adapter-based “universal” CAR platforms, BiTE-mediated T-cell redirection, and tumor-retagging methods that introduce CAR-recognizable antigens onto tumor cells.

### 3.1 Multi-target CAR designs

Multi-target CAR-T therapies address tumor heterogeneity by simultaneously targeting multiple antigens. Among these approaches, tandem CAR-T cells (TanCARs) achieve multi-antigen recognition by incorporating two distinct antigen-binding scFvs within the extracellular domain of a single CAR construct (47). These systems typically operate through OR-gate Boolean logic, in which engagement of either antigen is sufficient to trigger T-cell activation. Across hematologic malignancies and solid tumors, TanCAR designs targeting antigen pairs such as CD19/CD20, CD19/CD22, HER2/IL-13Rα2, IL-13Rα2/EphA2, CD70/B7-H3, MUC1/PSCA, FRα/mesothelin, and NKG2D ligands/CLDN18.2 have shown the potential to broaden antigenic coverage and reduce antigen-loss escape (48–59). In glioblastoma and other solid tumor models, these approaches generally improved tumor recognition and antitumor activity compared with single-targeted CAR-T cells, while early clinical evaluation of selected TanCAR products has also begun (54–59).

Multi-CAR-T cells are engineered to co-express two or more independent CAR molecules with distinct antigen specificities, enabling a single T cell to recognize multiple tumor antigens through physically separate receptor structures. This configuration can be generated either through co-transduction with separate CAR vectors or through bicistronic and multicistronic expression systems (60–62). In glioblastoma, dual- or trivalent CAR-T designs targeting combinations such as HER2, IL-13Rα2, EphA2, and EGFR have broadened antigenic coverage across heterogeneous tumors and have shown enhanced functional activity compared with monospecific approaches (62–64). Similar bicistronic strategies have also been extended to other solid tumors, including fibroblast growth factor receptor 4 (FGFR4)/CD276-targeted CAR-T cells in rhabdomyosarcoma models (65). Together, these studies support multi-CAR architectures as a means to address heterogeneous antigen expression, although receptor configuration, manufacturing complexity, and potential additive toxicity remain important translational considerations.

Cocktail CAR-T cell therapy refers to the combined use of separate monospecific CAR-T products, each directed against a different antigen (66–68). Although this approach is more expensive and operationally complex because it requires parallel manufacturing of separate cell products, it allows each CAR-T population to be individually optimized and delivered either simultaneously or sequentially, offering greater flexibility for addressing heterogeneous antigen expression patterns. Clinical and case-based experience with sequential or combined CAR-T products, including CD19/CD22-directed strategies in B-cell malignancies and EGFR/CD133-directed approaches in cholangiocarcinoma, supports the potential flexibility of this approach but also highlights added manufacturing and safety challenges (66–68). Overall, multi-target CAR-T strategies, including tandem CAR, multi-CAR, and cocktail approaches, have demonstrated substantial potential to overcome antigen heterogeneity and prevent tumor escape across hematologic and solid malignancies, although broader clinical implementation remains challenged by complex manufacturing requirements, limited *in vivo* persistence, and optimal antigen-pair selection.

### 3.2 Bispecific T-cell engagers in combination with CAR-T cells

Bispecific T-cell engagers (BiTEs) are engineered fusion proteins composed of two different scFvs that physically bridge T cells and tumor cells, thereby promoting formation of the immunological synapse and inducing T-cell–mediated cytotoxicity (69). Clinically, several FDA-approved T-cell–redirecting bispecific therapies, including blinatumomab (70), teclistamab (71), elranatamab (72), mosunetuzumab (73), and epcoritamab (74), have demonstrated substantial efficacy in hematologic malignancies. In solid tumors, this class of T-cell-redirecting therapy has also made meaningful clinical progress, with two agents currently approved. Tebentafusp, approved in 2022, recognizes a gp100-derived peptide presented by HLA-A*02:01 and is used for HLA-A*02:01-positive metastatic uveal melanoma (75). Tarlatamab, approved in 2024, targets DLL3 and is indicated for extensive-stage small cell lung cancer (76). Additional BiTE-based therapies are also under clinical investigation in solid tumors. For example, acapatamab, formerly known as AMG 160, is a PSMA-directed BiTE that has shown promise for metastatic castration-resistant prostate cancer (77), while the CEA-targeting CD3 bispecific antibody RO6958688 demonstrated antitumor activity in a phase I study of CEA-positive solid tumors (78).

Combining BiTEs with CAR-T cells has become an attractive approach for broadening target recognition and counteracting antigen heterogeneity (69, 79, 80). In one strategy, FRα-targeted CAR-T cells were combined with an oncolytic adenovirus delivering EGFR-targeted BiTEs (OAd-BiTE), enabling both CAR-T cells and endogenous bystander T cells to attack tumor cells, including those lacking FRα expression (81). Leveraging fourth-generation CAR designs, Choi et al. generated EGFRvIII-specific CAR-T cells capable of secreting EGFR-targeted BiTEs (CART.BiTE). This approach effectively controlled heterogeneous glioblastoma while avoiding detectable systemic toxicity following intracranial administration (80). Similarly, IL-13Rα2-directed CAR-T cells engineered to locally release EGFRvIII-targeting BiTEs were evaluated in glioblastoma models. In preclinical glioma models, these BiTE-secreting CAR-T cells achieved more effective early tumor suppression than dual-target CAR-T cells co-expressing IL-13Rα2- and EGFRvIII-specific CARs, although this advantage was not maintained long term (82). Beyond glioblastoma, GPC3-targeted CAR-T cells engineered to secrete B7-H3-directed BiTEs were shown to enhance the killing of hepatocellular carcinoma cells *in vitro*, including GPC3-negative/B7-H3-positive tumor cells, suggesting their potential to mitigate antigen heterogeneity-driven escape (83). A related strategy expanded BiTE-secreting CAR-T cells to recognize both cell-surface and intracellular tumor-derived targets. In this setting, Muc16-targeted CAR-T cells secreting a TCR-mimic BiTE specific for WT1/HLA-A2 showed enhanced antitumor activity against epithelial ovarian cancer cells with low Muc16 levels, both *in vitro* and in mouse models (84). Importantly, this general strategy has shown early clinical potential. Recently, in a first-in-human study, intraventricular administration of CARv3-TEAM-E T cells, which combine EGFRvIII-directed CAR recognition with local release of a T-cell–engaging antibody molecule directed against wild-type EGFR, induced rapid radiographic tumor regression in patients with recurrent glioblastoma. Although the responses 7were transient in two of the three patients, no dose-limiting toxicity was observed (85).

### 3.3 Switchable modular CAR-T platforms

Another approach for mitigating tumor heterogeneity is the development of modular CAR-T platforms, which allow one engineered CAR-T population to recognize different antigens through adapter molecules (86). In principle, this platform is highly flexible, as tumor cell killing can be achieved without re-engineering the T cells themselves. Instead, CAR-T cells can be directed toward different target cells simply by switching the administered adapter molecules. Modular CAR-T platforms not only enable multi-antigen targeting but also provide an additional layer of safety control, as CAR-T-cell activity can be modulated by titrating the adapter dose or rapidly terminated upon adapter withdrawal (87). Several adapter formats have been developed for modular CAR design, including biotin-based systems (88), fluorescein isothiocyanate (FITC)-based systems (89), and general control nondepressible-4 (GCN4)-binding CARs (90, 91). In these systems, cytotoxicity depends on the delivery of tumor-targeting antibodies carrying the corresponding biotin, FITC, or GCN4 tags (92). However, early adapter-regulated CAR platforms still face several design-related challenges. Non-native adapter-binding modules may raise immunogenicity concerns, while efficient T-cell engagement often requires empirical optimization of adapter architecture, including molecular size and spatial geometry, to enable productive immune synapse formation and robust CAR signaling (92).

To address some of these limitations and further improve the controllability of modular CAR-T platforms, next-generation programmable systems have been developed, including universal CARs (UniCARs) and split, universal, and programmable CARs (SUPRA CARs). The 5B9-based UniCAR system, developed by Bachmann and colleagues, is a representative modular CAR platform consisting of two components: (i) a signaling module, in which T cells express an anti-E5B9 scFv–based CAR, and (ii) a tumor-targeting module, in which a tumor-binding moiety is fused to the E5B9 peptide epitope originating from the human La/SS-B autoantigen (93). Importantly, this epitope has been extensively evaluated and shown to be non-immunogenic at both the T- and B-cell levels (92). This modular architecture further enables the same UniCAR-T-cell product to be redirected to diverse tumor antigens by exchanging the targeting module. Proof-of-concept studies have demonstrated UniCAR retargeting against multiple hematologic and solid tumor-associated antigens, including CD19, CD33, CD123, PSCA, PSMA, GD2, EGFR, and STn, highlighting its potential utility for addressing antigen heterogeneity through adaptable or combinatorial targeting strategies (93–99). Another programmable modular platform is the SUPRA CAR system, where T cells carry a universal leucine zipper receptor, termed zipCAR, that can engage tumor-targeting leucine zipper–scFv adaptors, termed zipFvs (20). The use of engineered leucine zipper pairs further enables programmable receptor–adaptor interactions, providing a basis for switchable and logic-gated regulation of CAR-T-cell function (100, 101). Experimental studies showed that the same engineered zipCAR T-cell population could be redirected toward distinct antigen-positive tumor cells using zipFvs targeting HER2, Axl, or mesothelin (20). Moreover, co-administration of anti-Axl and anti-HER2 zipFvs enabled effective killing of tumor cells expressing either antigen, thereby implementing an OR-gate targeting strategy and highlighting the potential of SUPRA CARs to mitigate antigen-heterogeneity–driven tumor escape without further genetic modification of the T cells (20). Beyond antigen retargeting, SUPRA CAR activity can also be tuned by modulating zipFv dose or leucine zipper affinity, allowing adjustable control over cytokine release and cytotoxicity (20). Despite these advantages, the clinical application of modular CAR-T platforms faces additional translational challenges related to repeated adaptor administration. The pharmacokinetic properties of adaptor molecules, including their half-life, biodistribution, and clearance kinetics, directly influence dosing regimens as well as the intensity, duration, and reversibility of CAR-T-cell activity (102). In the UniCAR system, small-sized target modules with short half-lives enable rapid T-cell silencing upon dose withdrawal, but may require continuous infusion for sustained activity (103). To address this limitation, IgG4-Fc-containing target modules with extended half-lives have been developed, allowing less frequent administration while maintaining antitumor efficacy (104). A staged approach combining short-lived modules for early safety control and long-lived modules for maintenance therapy has been proposed to optimize both safety and convenience (103). Furthermore, repeated dosing of exogenous adaptor proteins raises immunogenicity concerns, as anti-adaptor antibodies could neutralize adaptor function or accelerate its clearance (105). Humanization of adaptor components has been explored to mitigate this risk in SUPRA CAR systems (20). Collectively, these considerations underscore that switchable modular CAR-T platforms provide a flexible framework for addressing tumor antigen heterogeneity, but their clinical translation requires careful optimization of adaptor half-life, dosing convenience, tumor-targeting efficiency, reversibility, and immunogenicity to achieve sustainable long-term efficacy.

### 3.4 Antigen delivery and engineering strategies

In contrast to conventional approaches that primarily focus on identifying endogenous target antigens, antigen delivery and engineering strategies aim to introduce CAR-recognizable antigens into tumors, thereby broadening CAR-T recognition across antigen-heterogeneous or antigen-negative lesions (106–108). Among these approaches, oncolytic viruses (OVs) can be engineered to deliver exogenous CAR targets or expose virus-derived antigens on infected tumor cells. Thymidine kinase–deficient recombinant vaccinia viruses, including OV19t and mCD19-expressing vaccinia viruses, have shown that virally delivered CD19 can sensitize tumors to CD19 CAR-T-cell recognition and reduce antigen-low escape (109, 110). Building upon single-antigen tagging strategies, more advanced OV platforms, such as the dual-antigen oncolytic herpes simplex virus (oHSV) T7011, have been developed to broaden antigen coverage and combine antigen retagging with microenvironmental modulation through immune-modulating payloads (111). Beyond exogenous antigen delivery, viral proteins themselves, such as the vaccinia virus–encoded A56 protein, can also serve as CAR targets on infected tumor cells (112). Overall, OV-based approaches help overcome tumor antigen heterogeneity by introducing CAR-recognizable antigens into tumor cells while simultaneously leveraging viral oncolysis and immune activation, thereby enhancing antitumor efficacy and expanding the pool of targetable tumor cells.

In addition to viral retagging systems, several non-viral strategies have been developed to install synthetic CAR targets within tumors, either by directly modifying the tumor cell surface or by inducing local expression of synthetic antigens. These strategies can be broadly divided into direct membrane installation of synthetic targets and nucleic acid–based induction of *de novo* antigen expression. Among these, direct membrane retagging represents a particularly intuitive approach, as it bypasses the need for tumor-cell transcription or translation and instead endows malignant cells with exogenous CAR-recognizable ligands at the cell surface (113). Amphiphile tagging, pH-low insertion peptide fused to a c-Myc surrogate epitope (Myc-pHLIP), and fusogenic antigen-loaded nanoparticles represent membrane-engineering approaches that deposit exogenous CAR-recognizable ligands or antigens onto tumor-cell surfaces independently of native antigen expression (113–115). Whereas these approaches rely on direct installation of exogenous ligands or antigens onto tumor cell membranes, Gamboa et al. developed a genetically encoded retagging strategy, using lipid nanoparticles to deliver mRNA encoding an orthogonal VHH-based synthetic antigen into solid tumors (116). By inducing *de novo* surface antigen expression, this approach sensitized antigen-low, antigen-negative, and antigen-heterogeneous tumors to anti-VHH CAR-T-cell-mediated cytotoxicity, while reducing dependence on native tumor antigen profiles and mitigating antigen escape.

Beyond these material-based retagging strategies, bacteria have emerged as an alternative platform for tumor-targeted antigen delivery because they preferentially colonize hypoxic and necrotic tumor cores (117–119). Leveraging this property, engineered bacteria have been developed as intratumoral bioreactors that continuously produce therapeutic payloads and promote tumor regression (120–123). Although programmable bacterial platforms appear to be generally well tolerated in patients with solid tumors, their efficacy across broader indications remains to be established (124–126). Probiotic-guided CAR-T strategies, such as ProCAR, have shown that engineered bacteria can locally release synthetic CAR target antigens within tumors, activate antitumor immunity, and broaden CAR-T-cell recognition beyond endogenous antigen expression (127). Related bacteria-based approaches have also been extended to CAR macrophage combination strategies, in which tumor-colonizing bacteria locally release bispecific engagers to support recognition of antigenically diverse tumor cells (128). Nevertheless, the use of live bacteria may raise safety concerns related to bacterial translocation and persistent systemic survival (129, 130). Bacterial outer membrane vesicles (OMVs) are nanoscale vesicles released from Gram-negative bacteria and carry a broad range of bacterial constituents, making them attractive tools for cancer immunotherapy (131–133). Compared with live bacterial therapies, OMVs offer a safer cell-free format while retaining intrinsic immunostimulatory activity and the capacity for further engineering as delivery vehicles (134–137). More recently, a bacterial OMV-based platform termed BROAD-CAR was developed, in which OMVs were engineered to display an anti-PD-L1 antibody and carry plasmids encoding CAR-recognizable antigens. By remodeling tumor antigenicity directly within solid tumors, BROAD-CAR rendered antigen-heterogeneous and antigen-negative tumors susceptible to CAR-T-cell-mediated killing and suppressed tumor recurrence and metastasis in a murine breast cancer model (138). Despite these advances, the durability and spatial uniformity of synthetic antigen retagging remain important translational concerns, particularly in large solid tumors. In an oncolytic foamy virus model carrying CD19 as a CAR target, CD19 CAR-T-cell pressure was associated with escape tumors containing viruses with deletions in the delivered CD19 transgene, indicating that antigen loss from the viral vector can occur under CAR-T selection (139). For non-viral approaches, incomplete penetration into poorly vascularized or densely stromal tumor regions may lead to heterogeneous antigen deposition, a concern consistent with established delivery barriers in solid tumors that limit the uniform distribution of nanoparticles and other macromolecular agents (140). Such incomplete retagging could leave antigen-negative tumor foci intact, allowing immune escape. To address this, dual-antigen-encoding oncolytic viruses such as OVDual, which delivers both CD19t and EGFRvIIIt, have been developed to limit immune escape through broader antigen coverage (141). Clinical translation will thus require careful evaluation of antigen-expression durability, intratumoral distribution, residual antigen-negative regions, and delivery-system optimization to improve spatial uniformity of antigen installation.

## 4 Improving CAR-T cell trafficking and tumor infiltration in solid tumors

Efficient homing to malignant lesions and subsequent penetration into tumor tissue are essential prerequisites for CAR-T-cell efficacy in solid tumors. Improved tumor-directed trafficking may also enhance safety by reducing the required CAR-T-cell dose and limiting peripheral dissemination, where on-target, off-tumor toxicity may occur. Nevertheless, CAR-T-cell entry into solid tumors is frequently restricted by impaired chemokine gradients, discordant chemokine receptor expression, altered adhesion signaling, abnormal tumor vasculature, and extracellular matrix-rich stromal barriers (9, 142). To bypass inefficient recruitment from the peripheral circulation, locoregional administration represents a direct and promising strategy. Preclinical evidence has demonstrated that regional delivery significantly reduces tumor burden in models of mesothelioma (143), glioblastoma (144), breast cancer (145), colorectal carcinoma liver metastases (146), and pancreatic cancer liver metastases (147). Clinical experience in high-grade gliomas and malignant pleural mesothelioma further suggests that intratumoral, intracerebroventricular, or intrapleural delivery may improve tolerability and provide preliminary signs of antitumor activity in selected settings (148, 149). Biomaterial-assisted regional delivery, such as fibrin matrix–based CAR-T-cell implantation, may further enhance local retention and residual tumor clearance while limiting systemic exposure (150).

Another strategy to enhance the tumor-homing and infiltration capacity of CAR-T cells involves genetic engineering to overexpress specific chemokine receptors, thereby enabling these cells to sense tumor-derived chemokine gradients and execute directed migration (8). The therapeutic potential of this strategy was demonstrated in early preclinical studies showing that CCR2b-engineered GD2- or mesothelin-specific CAR-T cells exhibited enhanced tumor trafficking and improved antitumor efficacy in mouse models of neuroblastoma and mesothelioma, respectively (151, 152). Subsequent work by Huang and colleagues further showed that introducing CXCR1 or CXCR2, two receptors for IL-8/CXCL8, into CAR-T cells markedly increased their tumor infiltration and persistence, leading to complete tumor regression and durable antitumor immunity in aggressive tumor models such as glioblastoma, ovarian cancer, and pancreatic cancer (153). Beyond these examples, additional chemokine-axis engineering strategies have been explored to promote CAR-T-cell recruitment and retention within tumor lesions. These include ectopic expression of CXCR4, CXCR5, CXCR6, CCR8, CX3CR1, and CCR4, as well as engineered secretion of CCL19 or CCL21 (8, 154–159). Although chemokine receptor engineering can improve CAR-T-cell trafficking when tumor-derived chemokines are matched with their cognate receptors, its efficacy and safety depend on the biological specificity of the selected chemokine–receptor axis (8). Because chemokines regulate physiological leukocyte trafficking in normal tissues and lymphoid organs, ectopic receptor expression may redirect CAR-T cells toward non-tumor chemokine sources, resulting in aberrant homing, peripheral sequestration, and reduced tumor delivery (160). This risk is particularly relevant when the selected chemokine-receptor axis is not tumor-restricted or when systemically infused CAR-T cells show broad biodistribution outside tumor sites (161). In addition, chemokine expression within solid tumors is spatially heterogeneous and dynamically regulated, and dysregulated chemokine signaling can contribute to effector-cell exclusion and immunosuppressive-cell accumulation within the TME (162, 163). Thus, even with matched receptor expression, uneven intratumoral chemokine distribution may not guarantee deep tumor penetration and could instead retain CAR-T cells in peritumoral or chemokine-rich stromal regions (164). Chemokine receptors may also influence CAR-T-cell functional states beyond migration. For example, CXCR4 engineering has recently been shown to promote memory-associated transcriptional programs, reduce exhaustion, and support oxidative metabolism, indicating that receptor choice can shape CAR-T-cell differentiation and persistence (165). Therefore, chemokine receptor selection should integrate tumor chemokine profiles with off-tumor ligand expression, biodistribution risk, intratumoral chemokine organization, and potential effects on CAR-T-cell differentiation states.

CAR-T-cell infiltration and intratumoral accumulation are further impeded by the dense stromal architecture and disorganized vasculature characteristic of solid tumors. To overcome this barrier, considerable attention has focused on FAP, a stromal antigen highly expressed by cancer-associated fibroblasts (CAFs), which are major contributors to extracellular matrix deposition within the tumor microenvironment (166). Early studies demonstrated that combining FAP-specific T cells, which recognized and eliminated FAP-positive stromal cells through proinflammatory cytokine release and direct cytotoxicity, with EphA2-targeted T cells against A549 cancer cells conferred a synergistic antitumor effect and survival advantage superior to either monotherapy alone (167). A recent study engineered bispecific CAR-T cells co-expressing an anti-mesothelin CAR and secreting a FAP×CD3 T-cell engager, which demonstrated superior efficacy compared to monospecific controls in orthotopic mouse models of primary and metastatic liver PDAC (168). Despite these promising findings, systemic depletion of FAP-positive stromal cells raises important safety concerns. In two independent studies, FAP-targeted immune approaches eliminated FAP-expressing stromal populations in normal tissues, including skeletal muscle and bone marrow, leading to cachexia and anemia, or recognizing multipotent bone marrow stromal cells and inducing cachexia (169, 170). These findings indicate that FAP is not strictly tumor-restricted and that broad FAP depletion may produce on-target, off-tumor stromal toxicity. Therefore, FAP-targeting strategies may require localized, conditionally activated, or tumor-confined designs to improve the therapeutic window. For example, local delivery of FAP-targeted CAR-T cells was reported to be feasible and safe in an early phase I study of pleural mesothelioma (171). Similarly, mesothelin CAR-T cells secreting FAP×CD3 T-cell-engaging molecules may help concentrate FAP-directed stromal remodeling within mesothelin-positive tumor lesions by coupling engager release to tumor-directed CAR-T-cell localization, although systemic exposure and off-tumor FAP targeting still require careful evaluation (168). Beyond targeting CAF-associated stromal antigens, CAR-T cells can also be engineered to express extracellular matrix–degrading enzymes, including heparanase and human hyaluronidase PH20, thereby enhancing their ability to penetrate the dense tumor stroma (172, 173).

An alternative approach to improve CAR-T-cell entry into solid tumors is to target the abnormal tumor vasculature and promote vascular remodeling. Initial evidence supporting this concept came from adoptive transfer of T cells directed against VEGFR, a key angiogenic receptor, which inhibited tumor progression in several syngeneic mouse tumor models and human tumor xenograft models (174). Subsequent studies further demonstrated that CAR-mediated targeting of VEGFR-1 or VEGFR-2 expressed on tumor endothelial cells induced vascular disruption, facilitated CAR-T-cell entry into tumor tissues, and produced durable antitumor activity (175, 176). Other CAR-based strategies targeting tumor-associated vascular antigens include prostate-specific membrane antigen (PSMA) (177), αvβ3 integrin (178), TEM8 (tumor endothelial marker 8) (179), and EIIIB (180), an alternatively spliced fibronectin variant highly enriched in tumor neovasculature. Although CAR-T cells directed against tumor endothelial markers have shown consistent tumor-suppressive activity in preclinical models, murine studies have also raised safety concerns, revealing on-target off-tumor toxicity for some vascular targets (176, 181). In a phase I trial of a VEGFR2-specific CAR-T-cell therapy (NCT01218867), only 1 of 24 patients achieved a partial response, while the remaining patients did not achieve objective responses.

Recent studies have identified CLEC14A, a member of the C-type lectin domain family, as a promising tumor endothelial target because it is highly expressed on vascular endothelial cells in multiple common human cancers but shows low or undetectable expression in healthy tissues (182, 183). In preclinical models, CAR-T cells directed against CLEC14A suppressed tumor progression without detectable injury to normal tissues, supporting CLEC14A as a potentially safer vascular target for solid tumor immunotherapy (184). Similarly, Ash and colleagues developed E3K CAR-T cells, an endosialin/CD248-directed platform designed to eliminate CD248-expressing stromal elements. This antigen is enriched on tumor-associated pericytes and perivascular CAFs, but is only minimally detected in healthy tissues (185). Safety assessments in endosialin-knockout mice, tumor-free mice, and wound-healing models revealed no detectable antigen-dependent toxicity. At the same time, E3K CAR-T treatment depleted CD248-positive stromal cells, increased tumor necrosis, reduced tumor growth, and substantially impaired metastatic outgrowth in syngeneic breast and lung cancer models (185). Together, these findings indicate that effective CAR-T-cell infiltration into solid tumors will likely require coordinated optimization of delivery route, chemokine-guided migration, stromal remodeling, and vascular targeting, with safety evaluation remaining essential as trafficking-enhancing strategies move toward clinical translation.

## 5 Overcoming TME-mediated immune suppression

Once CAR-T cells infiltrate solid tumors, their effector function is frequently constrained by the immunosuppressive tumor microenvironment. Inhibitory checkpoint pathways, suppressive cytokines, regulatory immune cells, and metabolically hostile conditions can impair CAR-T-cell activation, cytokine production, cytotoxicity, and persistence (186–189). To overcome these barriers, recent CAR-based strategies have focused on blocking inhibitory signaling, enhancing intrinsic T-cell fitness, and remodeling the local immune milieu. These approaches include checkpoint-targeting strategies, armored CAR-T cells secreting immunostimulatory payloads, and synthetic receptor systems that rewire suppressive TME-derived signals into activating or pro-survival cues.

### 5.1 Counteracting checkpoint-mediated suppression of CAR-T cells

Immune checkpoint signaling, particularly the PD-1/PD-L1 and CTLA-4 axes, represents one of the best-characterized mechanisms by which the TME suppresses T-cell function. Accordingly, therapeutic approaches targeting these pathways mainly include combinatorial immune checkpoint blockade (ICB), genetic disruption or knockdown of checkpoint receptors, and local secretion of checkpoint-blocking antibodies or antibody fragments by engineered CAR-T cells (190–192). Clinically approved antibodies targeting CTLA-4, PD-1, PD-L1, and LAG-3 have been incorporated into the treatment of a broad range of malignancies, providing a strong rationale for combining ICB with CAR-T-cell therapy (193, 194). In preclinical models, such combinations, particularly those involving anti-PD-1 or anti-PD-L1 antibodies, have been shown to restore CAR-T-cell effector activity, alleviate exhaustion-associated dysfunction, and promote sustained antitumor immune responses (195–197). Clinically, a phase I trial evaluating intrapleural infusion of mesothelin-targeted CAR-T cells showed that subsequent treatment with pembrolizumab was well tolerated and clinically feasible, with evidence of antitumor activity in malignant pleural tumors (149). In addition to antibody-based ICB, genetic engineering approaches, including CRISPR/Cas9-mediated knockout and shRNA-mediated knockdown of checkpoint molecules, including PD-1 and CTLA-4, have been extensively explored to render CAR-T cells intrinsically resistant to checkpoint-mediated suppression and sustain their antitumor activity (198–201). Clinically, a phase I study (NCT03545815) of CRISPR/Cas9-engineered mesothelin-targeted CAR-T cells with PD-1 and TCR disruption demonstrated preliminary feasibility and safety in mesothelin-positive solid tumors. However, circulating CAR-T cells became undetectable beyond one month, suggesting that endogenous TCR signaling may be important for maintaining their persistence in solid tumor settings (202).

Another strategy is to engineer CAR-T cells for local intratumoral delivery of checkpoint-blocking antibody fragments, including anti-PD-1 scFvs, directly within the TME. For example, CAR-T cells secreting bioactive anti-PD-1 scFvs have been shown to strengthen T-cell activation under PD-L1-mediated inhibitory pressure and improve tumor suppression in solid tumor xenograft models (203). Similarly, PD-1 scFv-secreting EGFR-CAR-T cells, anti-PD-1 scFv/4-1BBL-secreting CAR-T cells, and CAIX-targeted CAR-T cells secreting full-length anti-PD-L1 antibodies have demonstrated enhanced tumor infiltration, persistence, or tumor-localized checkpoint blockade in different solid tumor models (204–206). Together, these studies support checkpoint-targeted modulation as an important approach for restoring CAR-T-cell activity in solid tumors, while also underscoring the need to optimize efficacy, persistence, and safety before broader clinical application. Nevertheless, although PD-1/PD-L1-centered strategies, including antibody blockade, genetic checkpoint attenuation, and local secretion of checkpoint-blocking antibodies or fragments, can restore CAR-T-cell activity, targeting a single inhibitory pathway may be insufficient in chronically stimulated solid tumor TMEs. Compensatory upregulation of alternative inhibitory receptors, particularly TIM-3, LAG-3, and TIGIT, has been documented following PD-1 axis inhibition, limiting therapeutic durability (207–209). Accordingly, next-generation checkpoint-remodeled CAR-T cells may require combinatorial strategies that simultaneously address the PD-1 axis and compensatory inhibitory pathways. Concurrent knockdown of PD-1, TIM-3, and LAG-3 via shRNA clusters enhanced antitumor efficacy in solid tumor models, with mechanistic studies showing that triple knockdown increased CD56 expression, thereby promoting CAR-T-cell infiltration, survival, and IFN-γ secretion (210). Similarly, simultaneous downregulation of PD-1, LAG-3, TIM-3, and TIGIT using a miRNA-based shRNA platform has been reported to improve CAR-T-cell activation, cytokine secretion, and persistence (211). Notably, different combinatorial pairs may yield distinct functional outcomes, as PD-1/TIGIT dual knockdown enhanced antitumor activity by combining improved short-term effector function with preservation of a less exhausted phenotype (201). Together, these studies indicate that multiplex checkpoint modulation may be required to overcome the multifaceted inhibitory barrier in solid tumors (212). Therefore, checkpoint-targeted CAR-T engineering should be considered a broader strategy that integrates PD-1/PD-L1 blockade with genetic attenuation, local payload delivery, and multiplex remodeling of compensatory inhibitory pathways.

### 5.2 Armoring CAR-T cells with immunostimulatory payloads

Another approach to counteract TME-driven immune suppression is the development of fourth-generation CAR-T cells, particularly armored CAR-T cells or T cells redirected for universal cytokine-mediated killing (TRUCKs), which are engineered to deliver immunostimulatory payloads that enhance CAR-T-cell potency and persistence while also engaging endogenous antitumor immunity. Early preclinical work on IL-12-armored CAR-T cells helped establish the TRUCK concept, showing that localized IL-12 release could activate innate immune responses against antigen-negative tumor variants and modulate immunosuppressive cells within the TME (213). In hepatocellular carcinoma models, GPC3-targeted CAR-T cells with NFAT-inducible IL-12 expression enabled antigen-responsive IL-12 delivery within tumors, which improved CAR-T-cell infiltration, persistence, and antitumor efficacy while reducing regulatory T-cell accumulation and potentially limiting systemic toxicity (214). More recently, CAR-T cells secreting a fusion protein composed of αPD-L1 and IL-12 achieved PD-L1-targeted, tumor-localized IL-12 delivery, thereby improving T-cell trafficking, tumor infiltration, TME modulation, and antitumor efficacy while reducing systemic inflammation-associated toxicity in prostate and ovarian cancer models (215). Clinically, this approach has entered phase I evaluation in recurrent ovarian cancer, as illustrated by MUC16 (ecto)-targeted CAR-T cells designed for IL-12 delivery and tested via intravenous and intraperitoneal administration to assess safety and preliminary efficacy (NCT02498912) (216).

Alongside IL-12, IL-15 has also been investigated as a clinically relevant cytokine payload designed to sustain CAR-T-cell expansion and functional persistence in solid tumors. In a clinical study of patients with GPC3-positive solid cancers, IL-15-armored GPC3-CAR-T cells exhibited enhanced *in vivo* expansion and improved intratumoral survival, leading to disease control in 66% of patients and antitumor responses in 33%. Nevertheless, the higher frequency of cytokine release syndrome emphasized the need for built-in safety-control strategies, such as cytokine blockade or inducible suicide switches (217). Another candidate cytokine is IL-23, a heterodimer composed of p19 and the IL-12 p40 subunit, which promotes memory T-cell proliferation and IFN-γ production, supporting its potential use as an immunostimulatory payload in armored CAR-T cells (218). A later study engineered CAR-T cells to express the p40 subunit, enabling activation-dependent autocrine IL-23 signaling that increased granzyme B expression, reduced PD-1 expression, and enhanced antitumor activity *in vitro*. Subsequent *in vivo* evaluation using xenograft and syngeneic solid tumor systems showed that p40-engineered CAR-T cells mediated superior tumor suppression compared with conventional CAR-T cells, with a more favorable safety profile than IL-18- or IL-15-expressing CAR-T cells (219). Beyond IL-12, IL-15, and IL-23, additional cytokine payloads such as IL-18, IL-21, and IL-7/CCL19 have also been incorporated into armored CAR-T designs to broaden their capacity to counteract the multifaceted barriers imposed by solid-tumor TMEs (220).

These encouraging findings notwithstanding, cytokine-armored CAR-T cells may also generate clinically relevant toxicity when cytokine release is excessive or insufficiently localized. Because pro-inflammatory cytokines can amplify both CAR-T-cell activity and endogenous immune responses, uncontrolled payload expression may trigger systemic inflammation, cytokine release syndrome (CRS), macrophage activation, endothelial injury, off-tumor immune activation, and organ damage (221, 222). For example, early clinical experience with MUC16ecto-targeted IL-12-secreting CAR-T cells in recurrent ovarian cancer (NCT02498912) showed that hemophagocytic lymphohistiocytosis, also described as macrophage activation-like syndrome, was observed at a high incidence when treatment was given after lymphodepleting chemotherapy (223). Similarly, clinical evaluation of IL-15-armored GPC3-CAR-T cells showed increased CRS incidence, with severe cases controlled by IL-1 or IL-6 blockade or activation of an inducible caspase-9 safety switch (217). IL-18-armored huCART19 cells also showed inflammatory toxicity in lymphoma, with CRS reported in 62% of patients and low-grade immune effector cell-associated neurotoxicity syndrome (ICANS) in 14% (224). Although IL-23/p40-engineered CAR-T cells displayed a more favorable preclinical safety profile than IL-18- or IL-15-expressing CAR-T cells, constitutive cytokine signaling in T cells and bystander immune-cell activation remain potential toxicity concerns (219). Beyond systemic toxicity, constitutive cytokine-expression systems may impose sustained proliferative and activation pressure on CAR-T cells, which could increase the risk of terminal effector differentiation, activation-induced cell death, or exhaustion-like dysfunction, whereas activation-inducible payload designs may provide more temporally restricted cytokine support (219, 225, 226). Therefore, successful translation of cytokine-armored CAR-T cells will require coordinated control of cytokine timing, localization, intensity, and reversibility. Strategies such as activation-inducible expression, membrane-anchored cytokine formats, dose optimization, cytokine blockade, and inducible suicide switches may help preserve antitumor activity while limiting systemic inflammation and treatment-related toxicity (214, 217, 227).

### 5.3 Engineering synthetic receptors to rewire inhibitory signals

**Figure 3:**
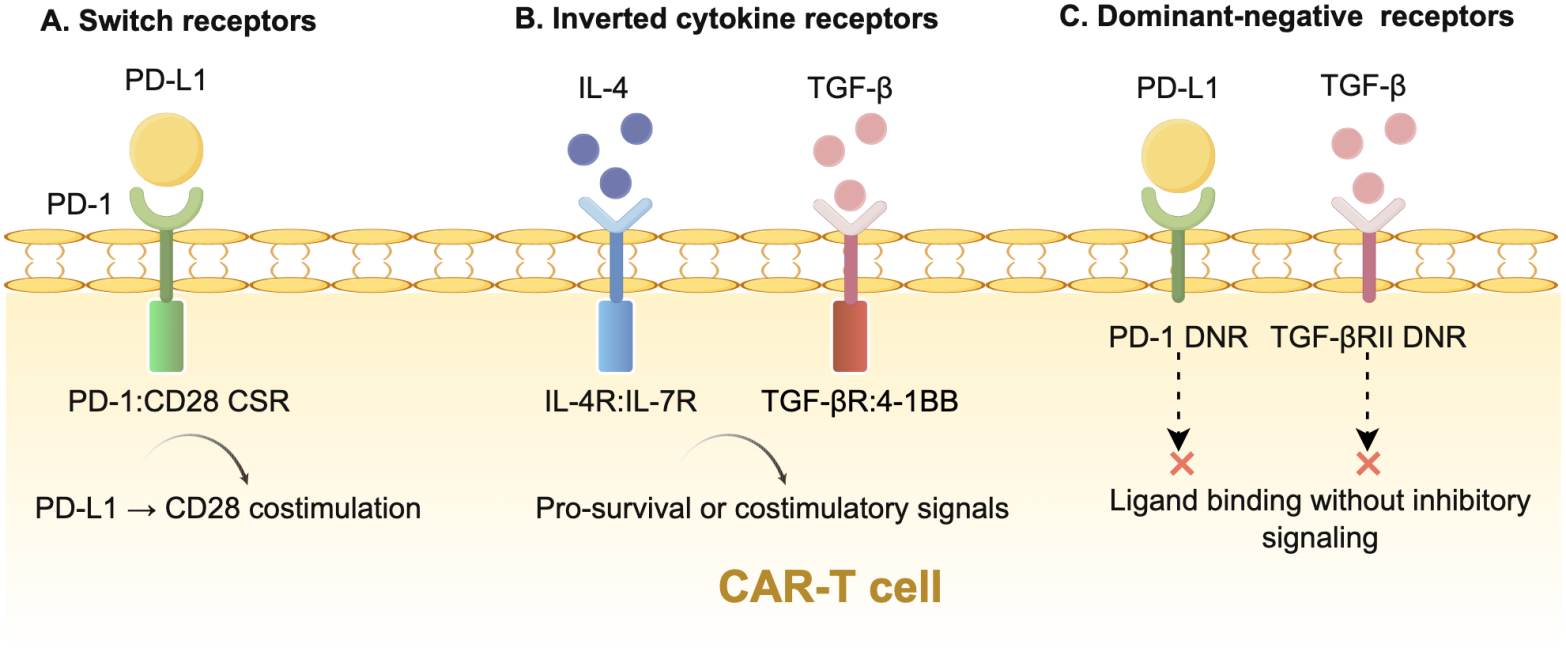
Synthetic receptors to rewire suppressive signals in the tumor microenvironment. **(A)** Chimeric switch receptors, exemplified by the PD-1:CD28 chimeric switch receptor (CSR), convert inhibitory PD-L1 engagement into CD28-mediated costimulatory signaling. **(B)** Inverted cytokine receptors, including IL-4R:IL-7R and TGF-βR:4-1BB receptors, redirect suppressive cytokine signals into pro-survival or costimulatory signals. **(C)** Dominant-negative receptors (DNRs), such as PD-1 DNR and TGF-βRII DNR, bind suppressive ligands without transmitting inhibitory downstream signaling, thereby shielding CAR-T cells from tumor microenvironment (TME)-derived immunosuppression. These receptor designs can restore CAR-T-cell function, enhance proliferation and persistence, and improve tumor-cell killing in solid tumors. The figure was created with Figdraw (https://www.figdraw.com).

Synthetic receptor engineering has emerged as a programmable approach to counteract TME-mediated suppression in CAR-T-cell therapy. As summarized in **Figure 3**, these strategies mainly include chimeric switch receptors, inverted cytokine receptors, and dominant-negative receptors, which either convert suppressive signals into activating or pro-survival inputs or prevent inhibitory downstream signaling. Chimeric switch receptors (CSRs) are designed to convert inhibitory checkpoint signals, including PD-1/PD-L1 signaling, into activating inputs that enhance CAR-T-cell function. In 2012, Michael C. Jensen and colleagues provided an early proof-of-concept for this strategy by developing a PD-1:CD28 CSR, in which the PD-1 extracellular domain retained PD-L1 recognition, while the transmembrane and intracellular regions were replaced by those of CD28 (228). This design converted PD-L1 engagement into CD28-mediated costimulation, enhancing ERK phosphorylation, cytokine secretion, proliferation, and granzyme B expression in primary human CD8^+^ T cells (228). In 2016, Liu and colleagues advanced this strategy by co-expressing a PD-1:CD28 CSR with mesothelin- or PSCA-targeted second-generation CARs, converting PD-L1 engagement into an additional CD28-mediated costimulatory input (229). In established mesothelioma and prostate cancer xenografts, PD-1:CD28-equipped CAR-T cells showed improved tumor regression, enhanced tumor infiltration, reduced tumor-induced hypofunction, and attenuated inhibitory receptor expression (229). Subsequent work in solid tumor xenograft models further refined this strategy by combining a universal PD-1:CD28 CSR with dual-targeting CD19/HER2 CAR-T cells. This design preserved antigen-dependent tumor killing while allowing PD-L1 engagement to selectively amplify CAR-T-cell proliferation and cytokine release, supporting the use of CSRs as modular costimulatory elements in PD-L1-rich solid tumors (230). In addition to rewiring checkpoint signals, CSRs have been adapted to counteract immunosuppressive cytokines in the TME, a strategy often termed inverted cytokine receptor (ICR) engineering. Early designs included IL-4-based ICRs that coupled the IL-4 receptor (IL-4R) ectodomain to intracellular signaling domains from IL-7R or the shared IL-2/IL-15Rβ chain, thereby converting IL-4 exposure into pro-survival or proliferative signaling (231–233). TGF-β-based ICRs have also been developed by linking the TGF-β receptor ectodomain to stimulatory intracellular modules. In a subcutaneous pancreatic cancer model, PSCA-targeted CAR-T cells co-expressing IL-4R:IL-7R and TGF-βR:4-1BB ICRs resisted IL-4- and TGF-β-mediated suppression and enhanced tumor-cell killing (234). In the clinical setting, intratumoral T4 immunotherapy (NCT01818323) has been tested for locally advanced or recurrent head and neck squamous cell carcinoma. This platform consists of pan-ErbB CAR-T cells equipped with an IL-4-responsive IL-4Rα:IL-2/15Rβ chimeric cytokine receptor and demonstrated manufacturability, persistence within injected tumors, no dose-limiting toxicities, and disease stabilization in 60% of treated patients (235). More recently, cytokine-switch designs have expanded beyond IL-4 and TGF-β, as illustrated by the G6/7R chimera, which links the extracellular domains of gp130 and IL-6Rα to an IL-7R signaling domain, enabling CAR-T cells to capture and degrade monocyte-derived IL-6 while enhancing their expansion, persistence, and antitumor activity in both hematologic and solid tumor models (236).

Co-expression of dominant-negative receptors (DNRs) provides a related but mechanistically distinct approach to resist suppressive TME signaling. Unlike CSRs or ICRs, which redirect inhibitory cues into activating inputs, DNRs competitively bind suppressive ligands without transducing downstream signals, thereby sequestering these ligands away from their native receptors (237). PD-1-based DNRs have been developed to provide cell-intrinsic checkpoint blockade, as shown by mesothelin-targeted CD28 CAR-T cells expressing a truncated PD-1 receptor in an orthotopic pleural mesothelioma model (200). This PD-1 DNR retained PD-L1 binding but lacked the intracellular inhibitory signaling domain, thereby restoring CAR-T-cell effector function and preventing PD-L1-mediated exhaustion within the TME (200). In parallel, TGF-βRII-based DNRs have become one of the most widely studied approaches for protecting CAR-T cells from cytokine-mediated suppression. Preclinical studies in prostate cancer showed that incorporating a dominant-negative TGF-βRII into PSMA-directed CAR-T cells conferred resistance to TGF-β-mediated inhibition and improved proliferation, cytokine secretion, persistence, exhaustion resistance, and antitumor activity, resulting in more effective tumor clearance in aggressive disease models (237). The same concept has moved into phase I clinical evaluation, where PSMA-directed CAR-T cells carrying a dominant-negative TGF-βRII are being tested in individuals with relapsed or refractory metastatic castration-resistant prostate cancer (NCT03089203), underscoring the translational promise of TGF-β-resistant CAR-T-cell strategies for solid malignancies (238). More recently, dnTGF-βRII armoring has been incorporated into a bicistronic EGFR/IL-13Rα2-targeted CAR-T platform for glioblastoma, where it enhanced T-cell proliferation and functional responses, improved bystander T-cell fitness, and strengthened antitumor efficacy in TGF-β-rich GBM models (239). Clinical data for C-CAR031, a GPC3-specific autologous CAR-T product incorporating a dominant-negative TGF-βRII module, showed a manageable safety profile and preliminary antitumor activity in heavily pretreated advanced hepatocellular carcinoma (HCC). In the phase I setting, C-CAR031 induced objective responses in 50.0% of treated patients (NCT05155189). Together, these synthetic receptor platforms provide complementary mechanisms to overcome TME-derived suppression by either rewiring inhibitory ligands into activating or prosurvival signals or blocking suppressive signaling at the receptor level, thereby supporting CAR-T-cell activity in solid tumors.

## 6 Emerging platforms for *in vivo* CAR-T cell generation

Most approved and investigational CAR-T cell therapies are generated ex vivo, requiring patient T-cell collection, genetic modification, expansion, product testing, and subsequent infusion. Although this manufacturing paradigm has produced substantial clinical benefit (240), it remains burdened by long production cycles, limited product shelf life, frequent dependence on lymphodepleting conditioning, and high manufacturing costs (241–243). *In vivo* CAR-T cell generation has therefore gained momentum as an alternative strategy, aiming to program endogenous T cells directly in the body through CAR-encoding nucleic acids or gene-editing cargo, while potentially simplifying treatment logistics and improving therapeutic performance (244). Recent advances in delivery technologies have expanded the toolkit for *in vivo* CAR-T cell generation, with major platforms including T-cell-targeted viral vectors, lipid nanoparticles, and other ligand-directed nanocarriers designed to deliver CAR-encoding genetic material to endogenous T cells (245).

Preclinical evidence suggests that *in vivo* CAR-T cell therapy can achieve effective tumor control while maintaining an acceptable safety profile. Among viral approaches, T-cell-targeted lentiviral platforms have provided some of the most advanced evidence for durable *in vivo* CAR-T generation. VivoVec-based systems, including UB-VV100 and its clinical successor UB-VV111, have demonstrated endogenous T-cell programming in preclinical models and have advanced toward early clinical evaluation in relapsed or refractory B-cell malignancies (246–248). Nevertheless, because lentiviral vectors enable durable gene transfer through genomic integration, their use for *in vivo* CAR-T generation requires careful assessment of vector biodistribution, target-cell specificity, off-target transduction, insertional mutagenesis risk, long-term CAR persistence, and the safety of sustained immune-cell engineering *in vivo* (244, 248). AAV-based systems have likewise been evaluated as alternative vehicles for *in vivo* CAR transgene delivery. Platforms such as AAV-DJ, CD8-directed DART-AAVs, and the engineered AAV6-M2 variant have shown the potential to improve T-cell tropism and reduce liver transduction in preclinical settings (249–251). However, AAV-based *in vivo* CAR delivery still faces challenges related to limited packaging capacity, pre-existing or treatment-induced anti-capsid immunity, incomplete T-cell specificity, and the need to better control transgene expression level and duration (252, 253).

In parallel, lipid nanoparticle (LNP)-based systems have emerged as a leading non-viral strategy for transient *in vivo* CAR-T cell programming (254). CD5-targeted LNPs loaded with CAR-encoding mRNA and antibody-targeted ionizable LNPs with extrahepatic tropism have demonstrated *in vivo* T-cell transfection and transient, tunable CAR expression without genomic integration (255, 256). JCXH-213, which uses LNPs to deliver CAR-encoding mRNA for relapsed or refractory B-cell non-Hodgkin lymphoma (NCT06618313), provides an early clinical precedent for non-viral *in vivo* CAR-T development. Selected clinical trials of *in vivo* CAR-T cell generation platforms for malignancies are summarized in **Table 2**. Together, these viral and non-viral platforms underscore the growing feasibility of *in vivo* CAR-T cell generation, although their broader application, particularly in solid tumors, will need further refinement in delivery specificity, biodistribution, expression control, repeat-dosing strategies, and long-term safety.

**Table 2:** Clinical trials of *in vivo* CAR-T cell generation platforms for malignancies.

Trial ID	Product	Delivery platform	Target antigen	Conditions	Status and phase
NCT06528301	UB-VV111 ± rapamycin	LV/VivoVec	CD19	R/R LBCL, CLL	Recruiting, Phase I
NCT06743503	UB-VV400 ± rapamycin	LV/VivoVec	CD22	R/R LBCL	Recruiting, Phase I
NCT06539338	INT2104	LV	CD20	R/R B-cell malignancies	Active, not recruiting, Phase I
NCT07075185	KLN-1010	LV	BCMA	R/R MM	Recruiting, Phase I
NCT06791681	ESO-T01	Immune-shielded LV	BCMA	R/R MM	Recruiting, Early Phase I
NCT06691685	ESO-T01	LV	BCMA	R/R MM	Recruiting, Phase I
NCT06678282 NCT06890065	JY231	LV	CD19	R/R B-cell lymphoma/leukemia	Recruiting, Phase I
NCT07002112	LVIVO-TaVec100	LV	CD19/​CD20	R/R B-cell malignancies	Recruiting, Phase I
NCT07395479	V001 Injection	LV	BCMA, GPRC5D, DLL3, FcRH5, by cohort	R/R advanced malignancies	Recruiting, Phase I
NCT06618313	JCXH-213	mRNA/LNP	CD19	R/R B-NHL	Recruiting, Phase I

LV, lentiviral vector; LNP, lipid nanoparticle; R/R, relapsed or refractory; LBCL, large B-cell lymphoma; CLL, chronic lymphocytic leukemia; MM, multiple myeloma; B-NHL, B-cell non-Hodgkin lymphoma.

Despite these advances, the clinical translation of *in vivo* CAR-T cell generation still requires careful control of delivery specificity and expression durability. After systemic administration, CAR-encoding vectors may distribute to or be taken up by multiple immune and non-immune compartments, making cell-type-resolved biodistribution and off-target delivery or transduction assessment essential for *in vivo* CAR-T platforms (246, 256). For integrating lentiviral vectors, durable genomic integration makes off-target transduction particularly concerning and requires evaluation of insertional mutagenesis, vector copy number, integration-site distribution, clonal expansion, replication-competent lentivirus, and long-term follow-up (257). AAV-based systems are generally non-integrating but still face limitations related to capsid immunogenicity, pre-existing neutralizing antibodies, liver tropism, limited repeat dosing, and rare integration or genotoxicity concerns (258). By contrast, mRNA/LNP platforms avoid genomic integration and enable transient CAR expression, but they may induce innate immune activation, complement-related inflammation, and repeat-dosing barriers (255, 259). Moreover, the short-lived nature of mRNA-based CAR expression may require repeated dosing and could limit efficacy in solid tumors that depend on sustained effector pressure for durable control (260). Therefore, the optimal *in vivo* CAR-T platform is likely disease- and context-dependent, reflecting the tradeoff between durable but integration-associated viral strategies and more controllable but less persistent non-viral mRNA/LNP approaches.

## 7 Conclusions

CAR-T cell therapy has achieved remarkable success in hematological malignancies, but its activity in solid tumors remains constrained by multiple interrelated barriers. The scarcity of truly tumor-specific antigens, together with heterogeneous or low-density target expression and antigen loss, can impair tumor recognition and promote immune escape. Even when target antigens are present, CAR-T cells often fail to reach and penetrate tumor lesions because of chemokine–receptor mismatch, abnormal tumor vasculature, and extracellular matrix-rich stromal barriers. Within the tumor bed, checkpoint signaling, suppressive cytokines and immune cells, and metabolic stress further limit CAR-T cell expansion, persistence, and cytotoxic function. These linked obstacles suggest that improving therapeutic outcomes in solid tumors requires more than enhancing CAR-T-cell killing capacity alone. Current engineering strategies therefore aim to improve tumor selectivity through logic-gated receptors, reduce antigen escape through multi-antigen, modular, and retargeting platforms, enhance tumor access through locoregional delivery and chemokine-receptor engineering, and counteract TME-mediated suppression through armored CAR-T cells, checkpoint-resistant designs, and synthetic receptors that convert inhibitory cues into activating signals. Emerging *in vivo* engineering approaches may further simplify CAR-T generation, but the controllability of transgene expression, durability of CAR expression, and long-term safety remain unresolved. Future studies should pair these designs with improved antigen discovery and more predictive models, including patient-derived organoids, organoid–immune cell co-cultures, patient-derived xenografts, and humanized mouse models, to better evaluate tumor recognition, CAR-T-cell infiltration, functional persistence, and safety before clinical testing.
